# Supplementary material for: Generation of annotated multimodal ground truth datasets for abdominal medical image registration
Source: Int J Comput Assist Radiol Surg. 2021 May 2;16(8):1277–85. doi: 10.1007/s11548-021-02372-7 (PMC8295129; doi:10.1007/s11548-021-02372-7)
Supplement: Supplementary file 1 — Supplementary material 1 (pdf 532 KB) [file 11548_2021_2372_MOESM1_ESM.pdf]

Supplementary Material

1 ASSD and DSC Registration Evaluation Metrics

To evaluate the proof of principle registration, we calculated the Dice similarity coefficient (DSC) in the manuscript:

$$\mathrm{DSC}(X,Y) = \frac{2\,|X \cap Y|}{|X|+|Y|},$$

where X are the registered liver masks and Y are the ground truth CT liver masks.
In this document we additionally provide the average symmetric surface distance (ASSD):

$$\mathrm{ASSD}(X,Y) = \frac{\sum\limits_{x \in X} \min d(x,Y) + \sum\limits_{y \in Y} \min d(y,X)}{|X|+|Y|},$$

In the manuscript we presented boxplots of the DSC shown in Sup. Figure 1. Here, we additionally show boxplots for the ASSD (Sup. Figure 2) and mean values for the DSC (Sup. Table 1) and ASSD (Sup. Table 2). The distributions in both boxplots are very similar and the outliers (failed registrations) stem from the same cases. The mean values shown in the tables show the same tendencies as the boxplots for ASSD and DSC. We therefore argue, that both metrics lead to the same conclusions regarding the quality of the registrations. Thus, we decided that only presenting the boxplot of the DSC in the manuscript is sufficient and avoids redundancy.

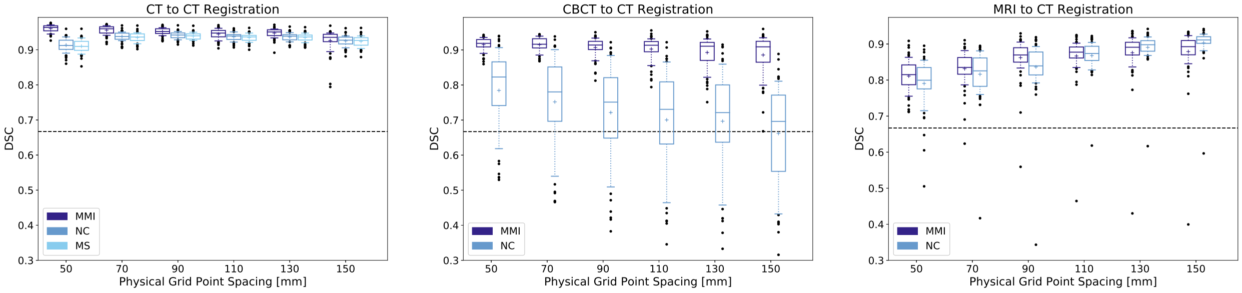

Sup. Figure 1: DSC for the proof of principle registrations with 56 data points each. The mean is marked as a "+" and the whiskers indicate the 10th and 90th percentile. All outliers are depicted as black dots. The dashed horizontal line shows the mean pre-registration DSC.

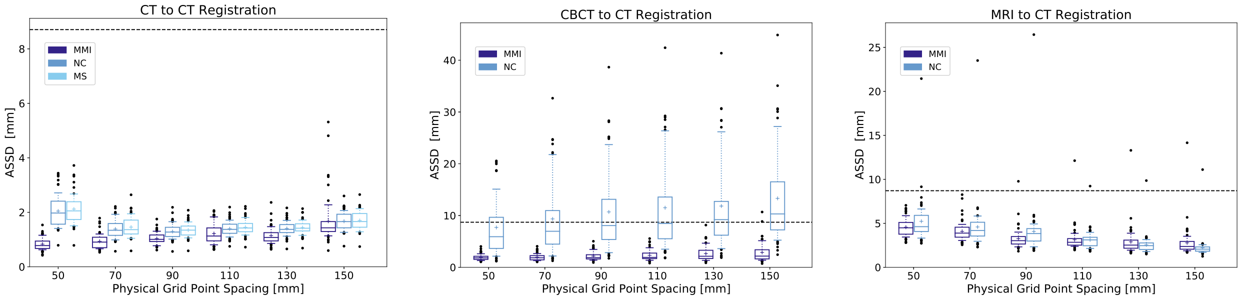

Sup. Figure 2: ASSD for the proof of principle registrations with 56 data points each. The mean is marked as a "+" and the whiskers indicate the 10th and 90th percentile. All outliers are depicted as black dots. The dashed horizontal line shows the mean pre-registration DSC.

Sup. Table 1: Registration evaluation DSC metric. The best result for each modality is highlighted. Mean pre-registration DSC: 0.667 ± 0.035

	DSC (CT to CT)					
Grid Point Spacing [mm]	50	70	90	110	130	150
MMI	$\mathbf{0.960 \pm 0.010}$	0.956 ± 0.013	0.952 ± 0.010	0.945 ± 0.015	0.948 ± 0.013	0.926 ± 0.031
NC	0.913 ± 0.020	0.938 ± 0.013	0.941 ± 0.010	0.937 ± 0.011	0.936 ± 0.012	0.925 ± 0.015
MS	0.910 ± 0.019	0.935 ± 0.013	0.939 ± 0.010	0.935 ± 0.011	0.935 ± 0.012	0.924 ± 0.015

	DSC (CBCT to CT)					
Grid Point Spacing [mm]	50	70	90	110	130	150
MMI	$\mathbf{0.915 \pm 0.019}$	$\mathbf{0.915 \pm 0.020}$	0.907 ± 0.028	0.903 ± 0.034	0.893 ± 0.045	0.886 ± 0.058
NC	0.784 ± 0.116	0.752 ± 0.134	0.721 ± 0.143	0.700 ± 0.148	0.697 ± 0.149	0.662 ± 0.139

	DSC (MR to CT)					
Grid Point Spacing [mm]	50	70	90	110	130	150
MMI	0.811 ± 0.045	0.832 ± 0.048	0.862 ± 0.054	0.867 ± 0.061	0.876 ± 0.068	0.879 ± 0.073
NC	0.791 ± 0.069	0.816 ± 0.070	0.836 ± 0.077	0.868 ± 0.043	0.891 ± 0.042	$\mathbf{0.903 \pm 0.044}$

Sup. Table 2: Registration evaluation ASSD metric. The best result for each modality is highlighted. Mean pre-registration ASSD: 8.7 mm

	ASSD (CT to CT)					
Grid Point Spacing [mm]	50	70	90	110	130	150
MMI	$\mathbf{0.8 \pm 0.2}$	0.9 ± 0.3	1.0 ± 0.2	1.2 ± 0.4	1.1 ± 0.3	1.7 ± 0.8
NC	2.0 ± 0.6	1.4 ± 0.3	1.3 ± 0.3	1.4 ± 0.3	1.4 ± 0.3	1.7 ± 0.3
MS	2.1 ± 0.5	1.5 ± 0.4	1.3 ± 0.3	1.5 ± 0.3	1.4 ± 0.3	1.7 ± 0.3

	ASSD (CBCT to CT)					
Grid Point Spacing [mm]	50	70	90	110	130	150
MMI	$\mathbf{1.9 \pm 0.6}$	2.0 ± 0.6	2.2 ± 0.8	2.3 ± 1.1	2.6 ± 1.5	2.9 ± 1.9
NC	7.7 ± 5.5	9.4 ± 7.2	10.7 ± 8.1	11.5 ± 8.7	11.8 ± 8.7	13.3 ± 9.0

	ASSD (MRI to CT)					
Grid Point Spacing [mm]	50	70	90	110	130	150
MMI	4.6 ± 1.0	4.1 ± 1.1	3.3 ± 1.2	3.1 ± 1.4	2.9 ± 1.6	2.8 ± 1.7
NC	5.2 ± 2.6	4.6 ± 2.8	4.1 ± 3.2	3.1 ± 1.1	2.5 ± 1.1	$\mathbf{2.2 \pm 1.2}$

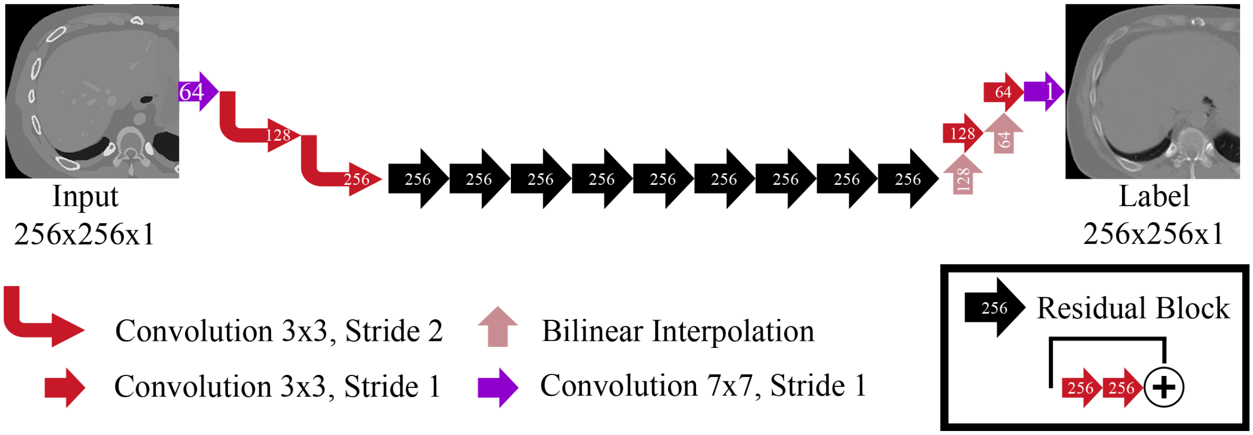

Sup. Figure 3: Res-Net architecture used for the CycleGAN generators. The numbers inside the arrows indicate the number of output channels of an operation.

2 ResNet network architecture

For the generators in the CycleGAN network, we used a Res-Net architecture with an encoding stage, 9 residual blocks and a decoding stage. A detailed schematic of the Res-Net is shown in Sup. Figure 3. In the encoding stage, the downsampling is performed via strided convolutions. The upsampling in the decoding stage is performed via a bilinear interpolation instead of a deconvolution, in order to avoid checkerboard artifacts. All convolutional layers use the Rectified Linear Unit (ReLU) activation function, except for the final convolution, which employs a hyperbolic tangent (tanh).
